# Commercial Applications of Metal Foams: Their Properties and Production

**DOI:** 10.3390/ma9020085

**Published:** 2016-01-29

**Authors:** Francisco García-Moreno

**Affiliations:** 1 Institute of Applied Materials, Helmholtz Centre Berlin, Hahn-Meitner-Platz 1, Berlin 14109, Germany; garcia-moreno@helmholtz-berlin.de or garcia-moreno@campus.tu-berlin.de; Tel.: +49-30-8062-42761; 2 Institute of Materials Science and Technologies, Technical University Berlin, Hardenbergstr. 60, Berlin 10623, Germany

**Keywords:** metal foam, metal sponge, production, properties, commercial application

## Abstract

This work gives an overview of the production, properties and industrial applications of metal foams. First, it classifies the most relevant manufacturing routes and methods. Then, it reviews the most important properties, with special interest in the mechanical and functional aspects, but also taking into account costs and feasibility considerations. These properties are the motivation and basis of related applications. Finally, a summary of the most relevant applications showing a large number of actual examples is presented. Concluding, we can forecast a slow, but continuous growth of this industrial sector.

## 1. Introduction

Metal foams are lightweight cellular materials inspired by nature. Wood, bones and sea sponges are some well-known examples of these types of structures. In fact, solid metallic foams are the conserved image of the corresponding liquid metallic foam. Scientific attempts to improve foam quality of any type of foam concentrate on the foam physics, *i.e.*, bubble formation, foam nucleation, growth, stability, development or gas diffusion in the liquid state, where the foam structure is evolving [1,2,3,4,5,6,7,8,9,10,11,12,13].

We have to distinguish between closed cell and open cell metal foams, which in fact should be called metal sponges more precisely speaking. The latter possesses also a foam-like structure, and therefore, we will consider the production, properties and applications from both classes. In contrast, other kinds of cellular or porous materials, like lattices, fibers, sintered granulates, honey combs, *etc.*, will not be taken into account, although they represent actually a larger market volume.

The engineering and industrial approach is to scale up the processes and provide reliable conditions for serial productions at acceptable quality and cost levels [14,15,16,17]. Depending on the production method, the foam structure is more or less homogeneous and comprises different characteristic features that determine its properties and, therefore, the fields of application [18].

Applications based on polymeric foams are well established in the market and part of our daily life. Some applications based on other foamed materials, such as porous concrete or food foams, are also quite popular in society, and several applications of ceramic foams are also well known in industry [19]. However, less common and familiar for us are applications and products based on metallic foams. The reason is that they are still not wide spread, although they have a very high potential, and a large number of applications already exist on the market. Some reviews about the applications of metallic foams are available in the literature [18,20,21,22,23,24]; however, in the last few years, new applications and application fields have emerged, and not all are considered commercially relevant.

A large number of companies made a bet on the future and started producing and commercializing metallic foams in the past two decades, although several have already closed, stopped production or concentrated on their core business, especially during the worldwide economic crisis starting 2007. Their major problem was caused not by the quality or properties of their products, but mainly by two factors: missing effective marketing for the small metallic foam market and the price, *i.e.*, their benefit margin. Some of the most relevant metal foam-producing companies at present are: Alulight (Ecka Granules, Mepura, Ranshofen, Austria), Cymat (Mississauga, ON, Cananda), Aluinvent (Miskolc, Hungary), M-pore (Mayser, Dresden, Germany), Pohltec Metalfoam (Collogne, Germany), Recemat (Dodewaard, the Netherlands), Exxentis (Wettingen, Switzerland), Alantum (Seongnam-City, Korea), Mott Corporation (Farmington, CT, USA), Foamtech (Daegu, Korea), Alveotec (Venissieux, France), ERG (Oakland, CA, USA), *etc*. A more detailed and regularly updated list can be found on the Internet at www.metalfoam.net [25]. Besides the companies’ names, some trade names have been established, being characteristic of the production method, such as Fominal (Frauenhofer, Bremen, Germany), Alporas (Shinko Wire, now Foamtech, Daegu, Korea), Alusion (Cymat, Mississauga, ON, Cananda), Aluhab (Aluinvent, Miskolc, Hungary), Duocel^®^ (ERG, Oakland, CA, USA), Incofoam (Inco, now Alantum, Seongnam-City, Korea), *etc*.

The main applications of metal foams can be grouped into structural and functional, and are based on several excellent properties of the material [18]. Structural applications take advantage of the light-weight and specific mechanical properties of metal foams; functional applications are based on a special functionality, *i.e.*, a large open area in combination with very good thermal or electrical conductivity for heat dissipation or as electrode for batteries, respectively. We will review the applications of closed, partially open and open cell metal foams. Open structures allow for media to penetrate through and provide interesting and wide-spread properties for functional applications. For serial applications, the production processes should be reliable and reproducible, and the final product should exhibit a reasonable price.

In the next paragraphs, we will first describe the commercial production procedures, then consider the most relevant properties of metal foams and discuss some other general properties and cost considerations. Both the production procedures and the unique properties are closely related to the possibility of an application to compete in the market at a reasonable price. Then, we will classify and review the most important fields of application and refer to some prominent examples of products and existing serial applications, but also of promising prototypes trying to explore further fields. In fact, a large number of applications are hard to classify into a certain category, as they are related to several properties. In this case, the classification will rely on the most relevant and characteristic property.

## 2. Commercial Production Procedures

**Figure 1:**
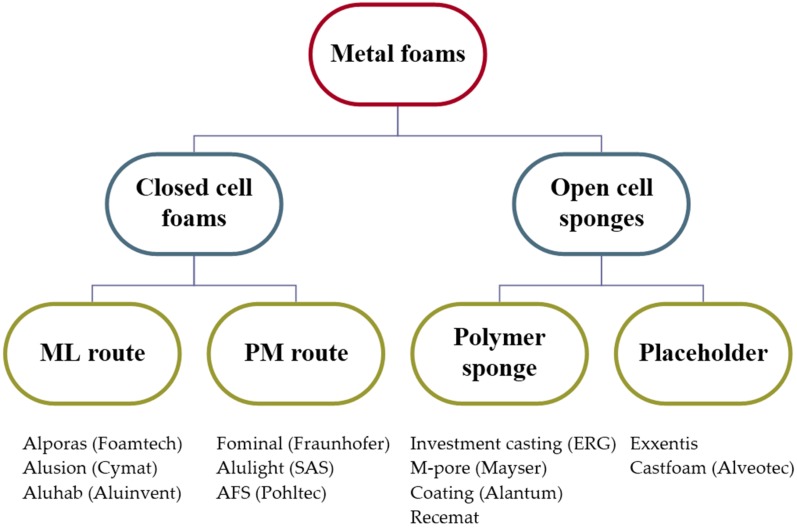
Schematic grouping of the commercially most relevant production methods of metal foams and sponges and some representative tradenames and companies. SAS, Slovak Academy of Sciences; AFS, aluminum foam sandwich; ML, melt; PM, powder metallurgical.

There is a large number of manufacturing processes for foams, sponges and porous materials with macroscopic pores. Here, we will review only the commercially most relevant methods. For closed cell foams, they are divided into two main families, also called manufacturing routes: the melt (ML) and the powder metallurgical (PM) route. They are also referred to as the direct and indirect foaming methods, respectively (see Figure 1). Most of these production procedures and their variations are already described in the literature [18,26,27]. Additionally, there are further manufacturing methods, especially applied in the case of metallic sponges, which can obviously not be foamed directly, as the gas will leak [28]. They are not directly connected with foaming in the strict sense of the word, but lead to a foam-like structure. These methods can be also divided into two main families: the first is based on a polymeric sponge structure as a pattern or carrier and the second on a placeholder that can be removed [22]. Their applications are usually focused on their functional character, but not only.

### 2.1. Melt Metallurgical Route

Shinko Wire, Amagasaki City, Japan, provided the first commercial production of metal foams in the late 1980s. In this process, calcium is first added to an aluminum melt and stirred in air to produce oxides and raise its viscosity. Subsequently, powder of the blowing agent (TiH_2_) is dispersed quickly into the melt by stirring, decomposing into gaseous hydrogen and titanium at the melt temperature. The melt starts then foaming inside the crucible or inside a dedicated mold. Finally, it is cooled down to a big block and usually sliced into plates of the desired thickness. This type of metallic foam and the corresponding process is called Alporas, which was patented in the United States of America in 1987 [29]. The patent is now expired, and the method has been used successfully by other companies and research centers, e.g., by Foamtech (Daegu, Korea) [30] or Shanxi Putai Aluminum Foam Manufacturing (Linfen, China) [31].

Another approach of direct foaming of an aluminum alloy melt was invented in the early 1990s by Alcan International Limited in Montreal (QC, Canada) [32], and Norsk Hydro (Oslo, Norway). In this process, the melt needs to be prepared with ceramic particles, such as silicon carbide or aluminum oxides, ranging from 5 to 20 vol% to increase the viscosity of the melt and to stabilize the liquid cell walls. Subsequently, air bubbles are injected and, at the same time, dispersed into the melt using rotating impellers. The bubbles rise to the top of the melt, where they are collected in a liquid foam and start solidifying after leaving the furnace, where the foam can be continuously drawn off using a conveyor belt. This method is used nowadays by Cymat (Mississauga, ON, Canada) to produce foam panels or to fill molds with foams that do not need further processing.

Aluinvent (Miskolc, Hungary) recently developed a technique to reduce the pore size and improve the pore size distribution of gas-injected foams using ultrasound oscillations to treat the melt during bubble formation. While the gas is introduced into the melt, sound waves induce earlier detachment of bubbles during their growing stage, which is not the case without this external force [33].

### 2.2. Powder Metallurgical Route

In the 1950s, Allen *et al.* patented a method for foaming metal very similar to the one used nowadays commercially [34]. This route consists of an indirect foaming of solid precursors by heating. The precursor is produced by mixing aluminum powders with the corresponding alloying elements and a blowing agent, typically 0.5 to 1.0 wt% of TiH_2_. Once the powder mixture is prepared, it is consolidated and sintered by extrusion, uniaxial compaction or rolling to yield a foamable precursor. By the heating of the precursors, the matrix starts melting, and the gas of the blowing agent nucleates. In the course of the temperature increasing, hydrogen production increases, and the gas diffuses to the nucleated pores, letting them grow into big bubbles and expanding the foam. The resident oxides in the metal powders (usually 0.5 to 1%) provide the stability of the foam during the holding time in the liquid state [35]. After several minutes, the foam development is fulfilled, and the foamed metal structure can be conserved by temperature reduction, leading to foam solidification.

In 1991, Baumeister *et al.* at Fraunhofer-Institute (Bremen, Germany) improved the method [36]. In the 2000s, Seeliger founded a new company, ALM (Saarbrücken, Germany) which become later part of Alulight (Ranshofen, Austria) and is known now as Pohltec Metalfoam (Collogne, Germany). Their product is an aluminum foam sandwich (AFS), which is an optimized lightweight panel of several square meters and thicknesses of 8 to 80 mm composed of a foam core and two bulk face sheets, both based on aluminum alloys. By this process, the metal powders and blowing agent are mixed and introduced inside a welded container. The container is then sealed and hot-rolled to a compact three-layer precursor, the container walls being the sandwich face sheets later. The advantage of this process is the metallurgical bonding between the face sheets and the foam core, obtaining a very stiff product with high temperature resistance that can be fully recycled [15].

### 2.3. Based on Polymer Sponge Structure

Metal sponges can be manufactured by replicating an open porous polymeric structure. Their morphology is based obviously on the structure of the polymeric foam, e.g., a polyurethane foam. This implies certain advantages, as their quality in the sense of pore homogeneity and distribution is superior, as polymeric foam technology is quite advanced and established compared to the metallic foam one. However, it has also several disadvantages concerning the number of production steps, dimensions, costs, *etc*.

An example is given by ERG (Oakland, CA, USA) which produces sponges of different metals, like aluminum or copper, known under the tradename Duocel^®^, following a foam replication or investment casting method. Here, the polymer sponge is filled with a slurry of heat-resistant material, e.g., a mixture of mullite, phenolic resin and calcium carbonate [37]. After drying under a certain temperature, the polymer is decomposed and the form stable enough for casting with the corresponding metal. The different grades are given in pores per inch (ppi), and porosities in the range of 80% to 97% are achieved.

A similar method, but with an additional wax covering of the polymeric foam for increasing stiffness, is used by Mayser GmbH, formerly M-pore (Dresden, Germany) [38]. The drying step with temperatures up to 350 °C allows one to remove the wax and the polymer, and metallic sponges with large and regular pores up to 10 mm in diameter can be produced.

Another method consists of a metallic coating of the polymeric sponge with subsequent sintering and removal of the polymer. An example is the deposition onto polyurethane foam by, e.g., chemical vapor deposition (CVD) of nickel tetracarbonyl (Ni(CO)_4_), which decomposes to elemental nickel and carbon monoxide at a heating temperature of 150 to 200 °C [39]. This method was developed and commercialized by Inco (Mississauga, ON, Cananda). Now, this technology is used by Alantum (Seongnam-City, Korea), with a large production plan in China [28,40], where they also produce iron and copper foams. Recemat (Dodewaard, the Netherlands) produces sponges of nickel, stainless steel, titanium, *etc.*, being metallized with the subsequent removal of the polyurethane sponge by pyrolysis. Generally, the initial metallization is done with nickel metal. The resulting nickel metal foam, after being heat treated, is used in applications where high electrical and thermal conductivity are of importance [41].

### 2.4. Based on Dissolution of a Placeholder

Metal sponges can be produced by infiltration or casting of molds filled with granulates. The latter have to be resistant to the high temperatures of the corresponding molten metal and be able to be removed by leaching or washing, like, e.g., salt or sand. The granules and their contact points correspond later to the pores and pore interconnections, respectively, leading to an open cell structure. The cell structure depends strongly on the granulate morphology. Special attention has to be given to the placeholder removal, to improve porosity and prevent corrosion.

Such types of aluminum alloy sponges in any types of forms are produced, e.g., by Exxentis (Wettingen, Switzerland) with pores in the range of 0.14 to 3 mm in diameter [42]. Similarly Alveotec (Venissieux, France), produces metal sponges, but usually with ordered structures [43]. They first produce an ordered core structure with a mix of silica and resin, then a preform together with the core and place them in a mold. Finally, they shed the metal into the grid and replace the preform after solidification.

Instead of casting around the placeholder, they can be mixed with metallic powders, compacted and sintered in a traditional PM way. In this case, not necessarily open cell structures are achieved, with different porosity ratios, depending on the nominal volume rate between powders and the placeholder. By the low amount of placeholder, these can stay isolated from each other, making their later removal almost impossible. This method is very interesting for the production of high melting alloys, such as titanium alloys. There, carbamide (urea) or ammonium hydrogen carbonate can be used as placeholders [44,45], but also sodium chloride [46].

## 3. Properties

Applications of metal foams are strongly linked to the properties that such kinds of materials can offer and especially to those that are excellent or even unique. Some of the properties are obviously mainly related to those of the matrix metal itself, e.g., elasticity, temperature or corrosion resistance, *etc*., while others appear only in combination with the cellular structure, e.g., low density, large surface area or damping. Therefore, the most remarkable types of mechanical, functional and other properties are reviewed here.

### 3.1. Mechanical Properties

The mechanical properties of metal foams are of course correlated to the ones of the corresponding bulk metal, but in a specific manner. The dominating factors here are the density and the structure itself. The foam structure is obviously the characteristic feature of a foam. Mechanical properties depend mainly on the density, but are also influenced by the quality of the cellular structure in the sense of cell connectivity, cell roundness and diameter distribution, fraction of the solid contained in the cell nodes, edges or the cell faces, *etc*. Gibson and Ashby proposed a simple beam model of a cubic cell for describing the mechanical response of foams [47]. Although this model is based on regular cellular structures, so-called lattices, it provides quite realistic results. Note that foam cells do not have eight neighbor cells, but usually around 14 [48]. Anyhow, this model and the experimental results show quite appropriately the strong dependence of the mechanical properties on the foam’s relative density [47,49,50,51,52,53]. The differences between open and closed cell foams can be comprehended in the sense of material distribution. Only in the case of a high velocity impact, the air captured in a closed cell foam has a notable additional contribution.

A certain mechanical property P^*^ of the foam should be evaluated in accordance with the weight, *i.e.*, density. Therefore, we should talk about high specific stiffness or moduli, or about mass-related properties, as P^*^ is usually not so good as the one for the bulk samples of the same volume *P_s_*, but there are exceptions, like damping, energy absorption, *etc.* Accordingly, the relative property is proposed in dependence of the relative density ρ^*^/ρ_s_, with the coefficient *a* and the structural constant *k*, as:

(1) $$\frac{P^{*}}{P_{s}}\approx k{\left(\frac{\rho^{*}}{\rho_{s}}\right)}^{a}$$

where *a* and the structural constant *k* depend on the property, on the foam’s structure and on the type of deformation.

One of the most important mechanical properties for structural applications is the stiffness or Young´s modulus. In our case, as already mentioned, we have to look for the relative modulus according to Equation (1). The Young´s modulus of a foam E^*^ corresponds to the slope of the compression strength *σ* in dependence of the strain *ε* in the elastic regime, *i.e.*,:

(2) $$E^{*}=\frac{\sigma }{\varepsilon }$$

Ashby [26] and Gibson *et al.* [47] proposed accordingly Equation (1) for the relative Young´s modulus:

(3) $$\frac{E^{*}}{E_{s}}\approx k{\left(\frac{\rho^{*}}{\rho_{s}}\right)}^{2}$$

where *E_s_* is the modulus of the solid bulk material; and *k* ≈ (0.1 to 4) in the low-density limit.

Based on their model, they further proposed a specific compressive strength of the foam in the elastic regime and came to the following approximation:

(4) $$\frac{\sigma_{el}^{*}}{E_{s}}\approx 0.05{\left(\frac{\rho^{*}}{\rho_{s}}\right)}^{2}$$

and similarly in the plastic regime:

(5) σpl*σys≈0.3(ρ*ρs)32

neglecting the density correction and the contribution of the entrapped gas in the case of closed cell foams, which plays a role only for high dynamic impact. σ^*^_el_ is the compressive strength in the elastic; and σ^*^_pl_ in the plastic regime, respectively; *σ_ys_* represents the yield strength. 

Considering the models and experimental results obtained by Gibson *et al.* [47], the compressive strength of an open cell foam in the plastic regime can be summarized to:

(6) σpl*σys≈k(ρ*ρs)32

and of the closed cell foam to:

(7) σpl*σys≈k[0.5(ρ*ρs)32+0.3(ρ*ρs)]

with *k* = (0.1 to 1.0) for both Equations (6) and (7).

A more detailed treatment of the models considering also further components in the case of compression, such as a density correction or a gas compression factor, can be found in the literature [47].

The tensile strength σ^*^_ts_ is a weak property of foams in general, as the resistance against a propagating crack mainly depends on the weakest link of the material, which is given here as a thin cell wall or Plateau border, which can break easily. σ^*^_ts_ is density independent and follows the relation:

(8) $$\frac{\sigma_{ts}^{*}}{\sigma_{ys}}\approx k\prime$$

with *k´* = (1.1 to 1.4) [26].

Evans *et al.* and Banhart showed that the bending stiffness of a flat panel of a given weight is inversely proportional to its density [18,54]. This is a special loading case, where foams can clearly show their potential.

A metal foam can suffer fatigue under compression or tension load, but mostly it is a combination of both, e.g., due to bending or torsion. Therefore, fatigue is mostly limited by the weakest property, namely by the tensile strength [55,56,57,58,59]. Many applications, especially in the automotive industry, suffer a large number of cyclic loads, and therefore, it is very important to guarantee no failure during the operative life time. Fatigue can be represented by the number of cycles that a foam can withstand at a certain load without structural degradation. As cracks grow in a catastrophic manner, a structural failure will be the result.

Most probably the best and most remarkable mechanical property of metal foams is their energy absorption capability [60,61]. Foams can absorb a maximum of mechanical energy without exceeding a certain stress limit *σ_D_* due to plastic, irreversible deformation over a large strain range. This property, usually isotropic, makes foams almost ideal crash absorbers. The maximum energy absorption per unit volume *W_max_* by the plastic deformation of a metal foam is the integral of the stress-strain curve up to the stress limit and given by Gibson and Ashby [47] as:

(9) WmaxEs=σDEs{1−3.1(σDEs)23}

The mechanical properties of metal foams can be improved, similar as for bulk metals, by different hardening mechanisms, such as alloy composition, grain refinements, heat treatments, *etc.* [57]. Furthermore, structural designs and property optimizations are possible depending on the application and practical aspects [60]. Furthermore, of course, heat or hardening treatments need to be compatible with the foaming procedure or foam stability.

Integral foam structures show a density and property gradient, usually with a denser skin and a lighter core, similar to a bone structure, but are difficult to manufacture [62,63]. These structures are bioinspired and can have accordingly better properties, like a homogeneous foam, like, e.g., bending stiffness. A further interesting optimization of a panel is the sandwich structure composed of two bulk face sheets and a lighter metal foam core, as will be presented later [15,64,65].

Concluding, we can say that the most remarkable mechanical properties of foams are their light weight, the relative strength in compression, the bending stiffness and the energy absorption, which are mainly exploited in the applications.

### 3.2. Functional Properties

**Figure 2:**
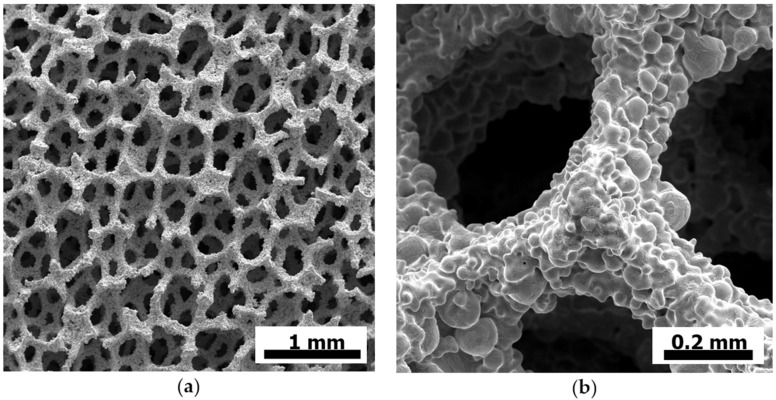
(**a**) Microscopic detail of the cellular structure of a NiCrAl sponge from Alantum; and (**b**) detail of the sponge surface (courtesy of Alantum).

There is a wide range of functional properties of metal foams originating from their cellular character. This structure implies a large surface area and number of cells (see Figure 2). Very often, the combination of functional properties, such as acoustic [66,67,68,69,70,71], thermal [72,73,74,75,76,77], electrical [21] or chemical resistance, with mechanical properties, like strength or stiffness, allows interesting new applications [54].

Foams in general are known for their thermal insulation properties, especially ceramic, glass and polymeric foams. They are the basis for a large number of applications related to thermal control. Thermal flow has to percolate through the matrix network of a foam structure; radiation between cell walls is hindered due to the large number of cells; and gas convection is disabled due to the small gas volume in each separated cell. Metal foams’ thermal conductance is less than that of the corresponding bulk metal, but of course, in absolute values, they cannot compete with ceramic or polymeric foams in terms of thermal insulation.

On the other hand, open cell metal sponges have a large surface area that can be used in combination with a good conductivity of the matrix metal (e.g., Cu or Al) for a completely different purpose, namely heat dissipation and passive cooling [75]. In fact, Al- and Cu-sponges can conduct temperature very well through the matrix, and therefore, such foams are used for heat exchangers. The relative thermal conductivity in metal sponges λ^*^/λ_S_ can be expressed in a simple model as a function of the relative density:

(10) $$\frac{\lambda^{*}}{\lambda_{s}}={\left(\frac{\rho^{*}}{\rho_{s}}\right)}^{a}$$

with *a* = (1.65 to 1.8) [26]. Further advanced models are summarized in the literature [77].

Metals are also known to be good electrical conductors. Again, the combination with a large sponge surface allows their utilization as electrodes in batteries or for other electro-chemical purposes. The electrical resistivity is given by:

(11) $$\frac{R^{*}}{R_{s}}={\left(\frac{\rho^{*}}{\rho_{s}}\right)}^{-a}$$

with *a* = (1.6 to 1.8) [26].

Polymeric foams are good for acoustic and vibration damping over a large frequency range due to the cellular structure, and the same property is present in metallic foams [67,70,78]. The latter can provide additionally electromagnetic shielding due to their metallic nature [15].

### 3.3. Other Properties

The foam structure and, accordingly, the foam properties are usually described as isotropic. However, obviously, the number of cells has to be large enough to be able to consider the foam as a continuum. This is why, e.g., for compression tests, the DIN Standard 50134 “compression test of metallic cellular materials” [79] requests that the number of cells in each direction of the sample tested has to be equal or larger than 10. Other cellular materials, e.g., lattices or honey combs, are obviously anisotropic in structure and, therefore, also in their properties. Anisotropy is an important advantage of metal foams for certain purposes, although some anisotropies in the structure can also be favorable for specific applications, and they also occur depending on the production method [21,80].

Recycling is becoming more and more important in our daily lives and is a required property for advanced and new materials. Obviously, the main component in a metallic foam is air, corresponding to 50 to 90 vol%. The rest is the metal matrix, which can be recycled as well as the metal material itself, among which aluminum alloy foams are the most prominent candidates. Additives, like the blowing agents (TiH_2_, ZrH_2_, CaCO_3_, *etc.*), are usually hydrides or oxides, which lose the gaseous component during the foaming process. The metal reacts with the matrix material and can be considered part of the alloy due to its metallic character and low amount (0.2 to 1 wt%) [81,82]. Although, there are also exceptions for recycling capability, such as gas-injected Al-foams stabilized by large amounts (5 to 20 vol%) of ceramic particles (usually SiC), commercially known as Cymat or Alusion foams [83].

The possibility to adjust the metallic foam density in a large range from 0.05 to 10 g/cm^3^ depending on the porosity and matrix metal allows for further properties, like buoyancy, which can be tailored varying the material density. This property can be interesting, e.g., for ships, floaters or sea markers, *etc.* It can be also combined with other properties, like high temperatures or corrosion resistance, where standard polymeric foams cannot be used.

Metals, like Ti or Mg, are bio-compatible or bio-degradable, respectively. These properties apply also to their foamed structures, being additionally able to adjust the Young´s modulus by adjusting their density and offering a large surface, especially in the case of metal sponges, for osseointegration, making medical applications self-evident.

## 4. Costs and Feasibility Considerations

A large number of successful prototypes have already demonstrated that there is a very wide range of possible applications. Depending on the desired performance and manufacturing feasibility, different production methods are considered. In most claimed applications, metal foams are as good or better than their competitors [18]. However, of course, one key point for industrialization breakthrough is still the price of the product [16].

Furthermore, and thinking about costs and system integration, bi-functional or even multi-functional applications are in favor [18,54]. Therefore, for real applications, cost-effective production methods and multi-functionality need to be combined as the priority goal [84]. A multi-purpose approach could be for example an application as a lightweight, stiff and recyclable material with vibration damping properties and very high volume-effective crash absorption protection for electric cars. In such cases, the balance between costs and benefits can decline to the side of metal foams. However, sometimes, other aspects, like innovation, image, marketing, prestige or strategical aspects, can play an important role. Depending on the application field, the price may then play a less important role, which is the case in the fields of the arts, design, medicine or sports.

More important even is to find applications where the properties provided by the metal foam are unique when compared to other materials and the price is only secondary, e.g., the best crash absorption behavior in a minimal space and weight to improve safety.

For mobility, *i.e.*, applications for the automotive, railway or aerospace industry, being light weight plays an enormously important role, as in these cases, saving weight leads to a large savings of energy, e.g., due to the continuous acceleration and breaking cycles of the mass. This point seems to be particularly important for actual electric cars and buses, where the batteries still do not provide a sufficient operation range.

Applications in aerospace could be the field where multi-functionality offers the most financial advantages. Thinking, e.g., about a plane fuselage, which is made traditionally from Al-sheets with welded or riveted struts, it could be replaced by a foamed, curved sandwich structure. A metallic foam sandwich will provide additionally noise and vibration damping of the turbines and a certain additional temperature insulation compared to the traditional metal sheets. It can be also recycled, and it is non-flammable. Saving 1 kg in a plane fuselage corresponds roughly to one million Euros in savings over the whole life of the plane (e.g., 25 years). Additionally, considering that demand and price for fossil fuels will increase strongly in the future, the advantages of lightweight materials will increase as well. However, the aeronautic industry seems still to have at present reservations against the application of metallic foams, most probably due to the long and expensive validation procedure of new materials and the actual trend toward fiber-reinforced composites.

For small or medium-sized applications, it is difficult to fix an actual price for metal foams, as it depends mostly on the metal, foam density, shape, degree of complexity, number of units of the desired product, *etc.* In the case of market-ready foams made by following the powder metallurgical route, the price of the raw material is ~3 €/kg for the Al-powder and <1 €/kg for the blowing agent (TiH_2_) (both related to the weight of the foam). In the last few years, attempts to reduce the price were focused on alternative blowing agents, such as CaCO_3_, as it almost represents 1/3 to 1/4 of the total price of the raw materials [85,86,87,88,89,90,91]. Unfortunately, these advances did not find commercial breakthrough in all types of manufacturing routes until now, due to the lack of properties [92]. However, anyhow, foam prices should not be given in price-per-weight, as this suggests an expensive product compared to the bulk material, but better in price-per-volume or price-per-area. As a rough estimation, we can find actually Al-foam sandwich panels on the market for prices in the range of 60 to 200 €/m^2^, depending on the type of foam, panel thickness, *etc*. A diagram of costs related to material density and modulus for different bulk and foamed metals can be found in the literature, although the given prices are no longer up to date [26].

## 5. Applications

### 5.1. Structural Applications

Lightweight materials with high stiffness are often desired for various applications. Standard products on the market, like honeycomb panels, use a cellular structure as the core and brazed or glued face sheets to provide the desired properties. They are usually inexpensive, but have some disadvantages, as they cannot be curved, resist high temperatures due to the glue nor be recycled. Aluminum foam sandwiches (AFS) and steel aluminum foam sandwiches (SAS) are promising products for structural applications and are already on the market [15,93,94,95].

AFS panels are used as support frames, e.g., for solar panels, mirrors, *etc.*, and everywhere where light and rigid metallic panels are needed. Most of the customers prefer to provide the material as panels and manufacture their own products themselves. Often they even keep their innovative field of application in secret to assure a competitive advantage for their products on the market.

**Figure 3:**
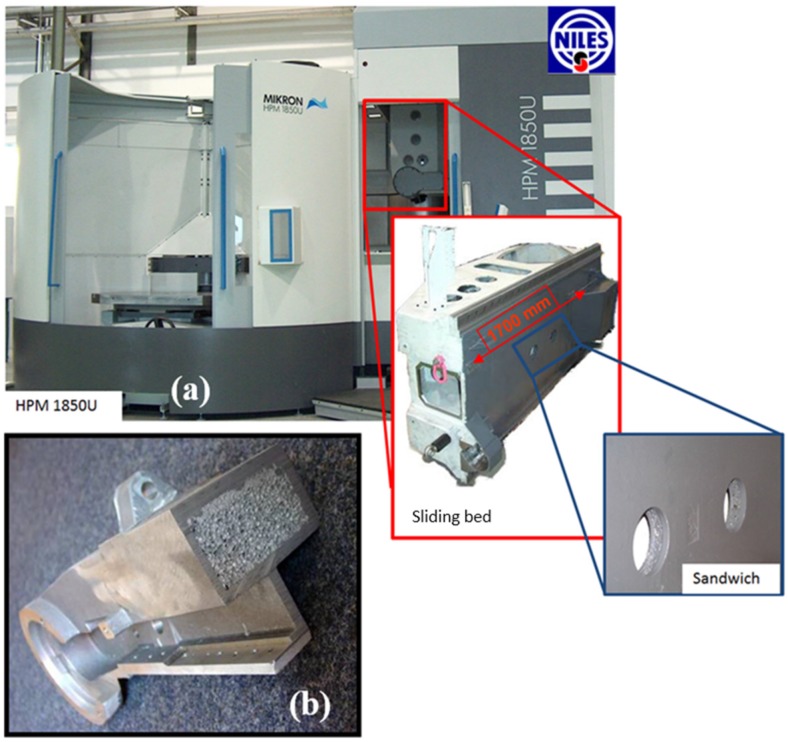
(**a**) Sliding bed of a milling machine made of welded aluminum foam sandwich (courtesy of Thomas Hipke, IWU, Chemnitz, Germany); and (**b**) a beam of a textile machine filled with Alporas foam (courtesy of the Au Metallgießerei, Sprockhövel, Germany).

A good example is industrial machines, where foam-filled beams and columns are stiff, but light. With reduced inertia, they can be moved quickly and positioned precisely. Examples are drilling, milling, textile, cutting, printing, pressing or blanking industrial machines. Additionally, damping of the system and of, e.g., an additional vibrating tool can improve the performance in the precision of positioning and wear, reduce fatigue problems and increase the operational lifetime. An example of an application for a high-speed milling machine of Niles-Simmons (Chemnitz, Germany) in cooperation with the Fraunhofer-Institut für Werkzeugmaschinen und Umformtechnik (IWU, Chemnitz, Germany) is given in Figure 3a. The sliding bed is made of 11 welded AFS parts, and the construction is 28% lighter than the cast part with the same stiffness, but improving vibration damping. Around 15 parts per year are manufactured. Figure 3b shows a beam of a textile machine filled with Alporas foam produced by the Au Metallgießerei in Sprockhövel, Germany. This part is 1590 mm × 280 mm × 160 mm and provides a 60% reduction in the amplitude at the resonance frequency. The production is ~1000 pieces per year. A similar hybrid material concept was applied to a tool column prototype of the Technical University Prague for a cutting machine (Model Prisma S) from TOS Varnsdorf s.a. (Varnsdorf, Czech Republic) in which an Alporas foam core is integrated.

**Figure 4:**
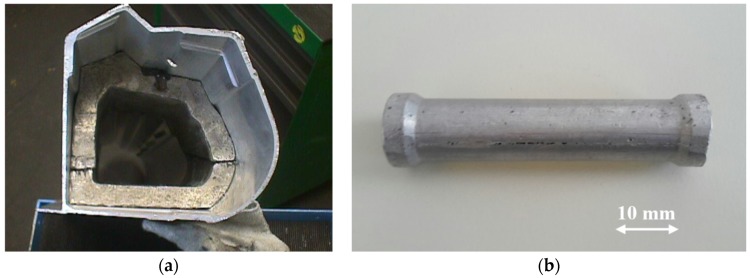
(**a**) Al-foam part for the Ferrari 360 and 430 spider; and (**b**) small crash-absorbing element for the Audi Q7 (courtesy of Alulight).

The automotive industry wants to benefit also from the developments of new light-weight materials. For large serial productions, not only material properties and costs are important, but also other engineering and strategical aspects play an important role, for example the necessity of the redesign of other components, the system integration capability or the number of available suppliers [96]. Besides a large number of prototypes, several large series went into production. As an example (Figure 4a), the door sill was filled with Al-foam to reinforce the frame and increase stiffness, as well as the behavior in the case of a side crash in high premium cars, like the Ferrari 360 and 430 Spider. The company Alulight (Ranshofen, Austria) holds a production from 1999 to 2009 with ~6000 pieces per year. The same company manufactured from 2006 another piece for the Audi Q7 SUV vehicle in a complete automated series, namely a small 7 g crash absorber (Figure 4b) placed at the support frame of the safety net [14]. With over 120,000 parts per year, this is until now the largest serial production of metallic foams in the automotive industry.

**Figure 5:**
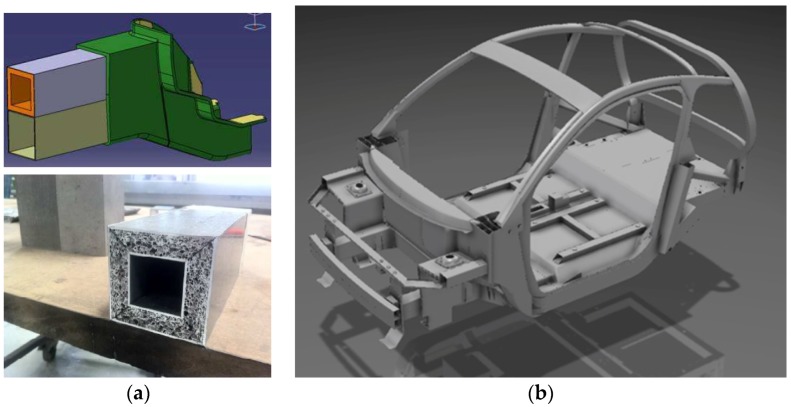
(**a**) Crash absorber box of the electric car prototype in the frame of the European project Evolution made with rectangular Al-profiles filled with Al-foam; and (**b**) CAD design of the body in white (courtesy of Cidaut, Valladolid, Spain, Pinningfarina, Cambiano, Italy and Pohltec metal foam, Collogne, Germany).

However, new opportunities for applications in the automotive industry offer new developments in the electric car segment, as there, lightweight construction becomes mandatory. Furthermore, new car designs are necessary due to the rearrangement of components, making it possible to consider cellular solutions from the beginning. Additionally, safety is another important factor, where in a reduced volume, a light, but very effective crash protection system is needed. An example is shown in Figure 5. Metal foam parts developed by the Technical University Berlin and Pohltec Metalfoam are foreseen in the prototype of an ultra-light electric vehicle developed in the frame of the European project Evolution [97].

Exploiting also the energy absorption capability of metallic foams, Foamtech (Daegu, Korea) provided crash elements for a guardrail at the Massan-Chanwon Bridge in Korea. Further, the suitability of Al-foam as a protection element in A-pillars was also analyzed in the literature [98]. An A-pillar of a Ford passenger car filled with Al-foam reinforcement provides an improvement of 30% in crash energy absorption at only a 3% weight increase [99]. A redesign of the components instead of a simple filling could even improve theses values.

**Figure 6:**
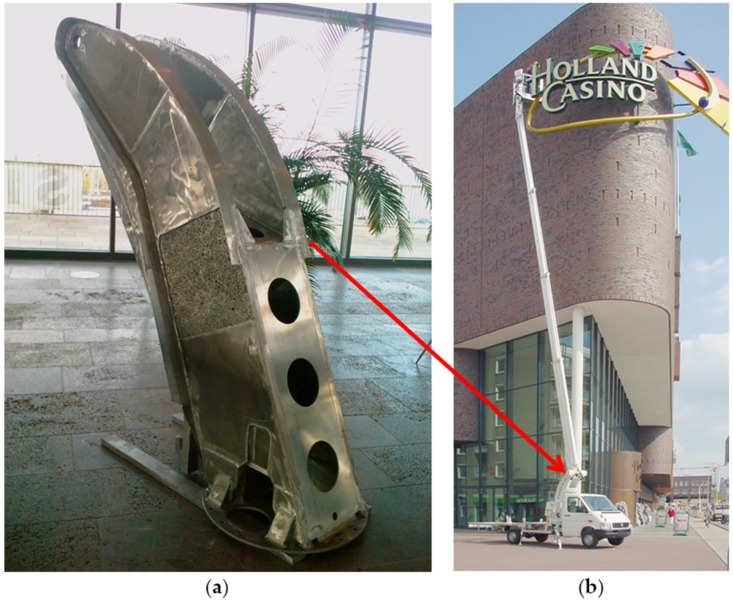
(**a**) Support of a working platform made of welded AFS manufactured by Pohltec Metalfoam; and (**b**) mobile crane vehicle from Teupen (Gronau, Germany) where the support is applied (courtesy of Pohltec Metalfoam).

Since 2003, Pohltec Metalfoam (Collogne, Germany), has been producing a support of a working platform made of welded AFS parts for mobile cranes for Teupen (Gronau, Germany) (Figure 6). This is a serial production of ~100 pieces/year, where the metallic foam component is saving around 95 kg compared to the original steel counterpart. This fact gives the company and the end user an important strategical advantage against competitors on the market, namely it is the only crane having a range of 25 meters and fulfilling the 3.5 ton weight limit to be driven with a normal driving license. AFS panels are also tested as lightweight support and crash protection of heavy battery modules at the bottom of electric car or bus bodies.

**Figure 7:**
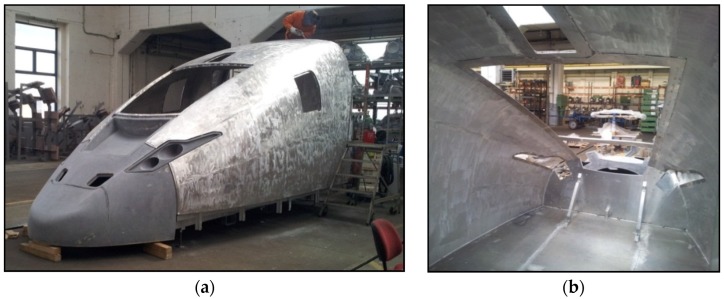
(**a**) Prototype of German high velocity train ICE made of welded aluminum foam sandwich; and (**b**) view of the interior with the low number of components required (courtesy of Thomas Hipke, IWU, Chemnitz, Germany and Voith Engineering, Chemnitz, Germany).

In the frame of mobility, we have to consider also the railway industry. Promising prototypes have evolved in the past years as possible future serial application. AFS foam panels delivered by the IWU (Chemnitz, Germany) are used in the floor of a wagon of the metro in Peking in continuous operation without issue for several years. A train front structure was welded from curved AFS plates by the Wilhelm Schmidt GmbH (Groß-Kienitz, Germany) in cooperation with the Brandenburgische Technische Universität Cottbus (BTU, Cottbus, Germany) [100]. A more prominent and recent prototype is the power head cover of the Intercity-Express-Train (ICE) train fabricated by Voith Engineering (Ludwigsfelde, Germany) and IWU (Chemnitz, Germany). It is made of welded AFS plates and carbon fibers in the front, with a total length of around 6 m (Figure 7). A weight reduction of 18% was achieved with the same stiffness, improving vibration damping and reducing additionally the manufacturing steps compared to the traditional construction procedure. These examples show clearly the advantages of metallic foams or AFS panels against honey comb panels, namely when curved sections are required.

**Figure 8:**
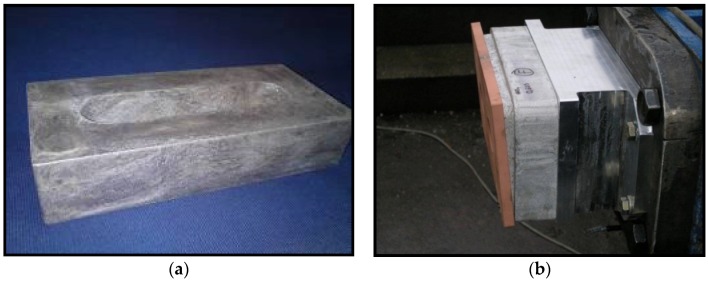
Crash absorbers (**a**) of the Combino tram in Budapest produced by Alulight; and (**b**) of the Sprinter Light Train in Holland produced by Gleich and SAS Bratislava (courtesy of Alulight, Gleich and SAS Bratislava).

Further railway applications can be mentioned, e.g., for the Combino tram in Budapest, where Alulight (Ranshofen, Austria) delivers a foam block placed behind the bumper to absorb energy in case of collisions with cars, trying to avoid expensive damage to the structure or write-offs (Figure 8a). A similar crash absorption box can be found in the Sprinter Light Train (SLT) in Holland, where ~1000 pieces per year were delivered since 2008 by (Gleich, Kaltenkirchen, Germany) and the Slovak Academy of Sciences (SAS, Bratislava, Slovakia) (Figure 8b).

**Figure 9:**
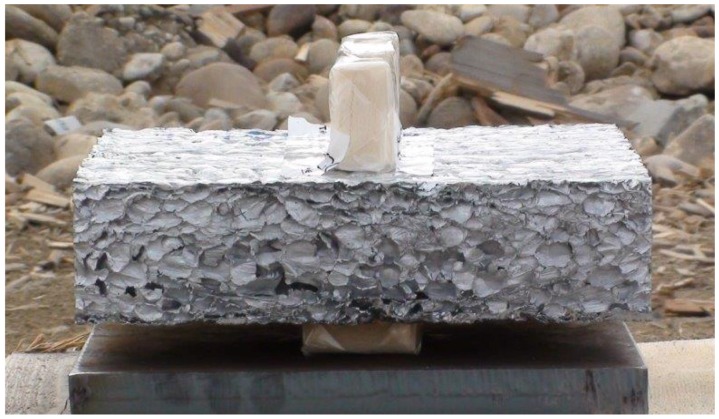
Block of Aluinvent continuous casted aluminum foam with an 85 mm thickness and 15 mm sized bubbles for a blast mitigation test with explosives shortly before detonation (courtesy of Norbert Babcsán, Aluinvent).

Crash absorption capability is of special interest for military applications related to armored protection of vehicles or blast mitigation. These types of applications are obviously mostly confidential, and it is difficult to gain detailed information about them. Metallic foams for protection and crash absorption, e.g., at the bottom of helicopters and of blast mitigation systems are good candidates. Figure 9 shows a foam block from Aluinvent with explosives to test its blast mitigation capability. Duocel^®^ aluminum foam provides critical blast energy absorption for crew protection in the cabin retrofit in the family of military medium tactical vehicles and in cabin seat mounts [37].

The ship building industry is considering metal foam applications concerning weight reduction and distribution, *i.e.*, upper structures should be as light as possible, to increase ship stability and, as a consequence, to increase ship load capacity. In 2009, a project concerning the development of a new type of inland area cargo vessels for weight reduction in ships started [101]. Furthermore, prototypes of sea markers made of metallic foam showed that in the case of damage due to ship contact or ice pressure, they still continue floating, being an important advantage for security reasons.

The aeronautic industry considered metallic foams for protection against bird strikes of planes [102]. Protection against micrometeoroids for satellites or in the space station are also of great interest, especially due to their isotropic properties [103,104]. Pohltec Metalfoam manufactured a prototype of a conical adapter (>4 m diameter) for the Ariane rocket with welded, curved AFS panels, performing better than standard materials due to additional vibration damping and demonstrating again the feasibility of curved and 3D structures.

For some sport equipment, the unique properties of metallic foams become more relevant, as price usually plays here a secondary role. For example, some golf putters use the damping properties of the metal foam structure to improve the strike control [21]. Some areas like shinbone protectors for football players or helmets could also be exploited. For example, Alcarbon (Bremen, Germany) combines the properties of aluminum foams with carbon fibers for sports articles [105].

### 5.2. Functional Applications

A wide palette of functional applications based on metallic foams can be found on the market. Again, a multi-purpose approach has the best chances to offer a competitive or unique product.

**Figure 10:**
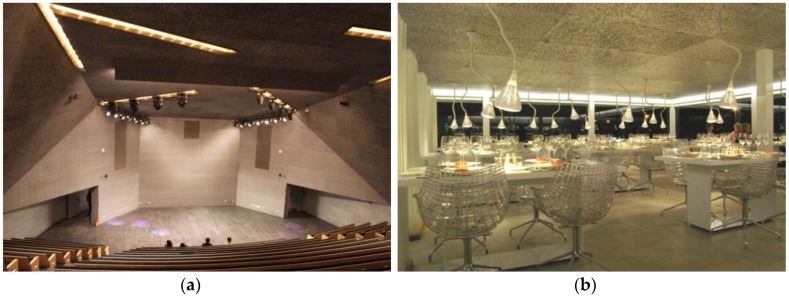
Ceiling of (**a**) audience hall; and (**b**) restaurant covered by Alusion foam for sound control (courtesy of Cymat).

Ceilings in auditoriums or large rooms are very often planked with perforated metal sheets for sound control. As an alternative to this traditional construction material, applications of metallic foam panels for sound absorption are already available on the market, offered by different companies [30,83,94]. At the open foam surface, the sound waves are guided and redirected to the foam interior, where they are caught and damped after several reflections. The pore size distribution and the different orientations allow for a very effective damping over a broad frequency spectrum. These applications could be considered also as architectural, but we include them here as their main function is sound absorption. They combine the advantage of the lightweight, self-supporting capability of large metallic foam panels made of open cell or just sliced closed cell foams, with a design component. Figure 10 shows the ceilings of an audience hall and a restaurant covered by Alusion foam provided by Cymat (Mississauga, ON, Cananda).

Further sound absorption applications made by Alporas foams provided by Shinko Wire (Amagasaki City, Japan) can be found in train rails, metro tunnels, elevators, on the under-side of an elevated highway, *etc*. [106]. Newer products based also on Alporas foams were developed by Foamtech (Daegu, Korea) and applied to concert halls, conference rooms, auditoriums, sport centers and walls and ceilings of the machinery and operating rooms of industrial plants as non-flammable, acoustic absorbers [30]. Further applications can be found in the ship industry, where, e.g., prevention of noise from the engine room, the combustive exhaust pipe and the air cleaning system are provided by foamed inside walls between cabins [30]. Metallic foams are also used by Foamtech for sound absorption in metro tunnels, in the railway and on the tunnel and station walls, where they have to support high air pressure changes and vibration, being at the same time non-flammable [30].

We can find aerodynamic noise reduction prototypes in the railway industry, e.g., in pantographs for the Shinkansen train in Japan [107,108]. There, open cell Al-foam is used for shape smoothing of the panhead and its support and covering. Applications for aerodynamic noise reduction of jet turbines of airplanes are also under discussion [109].

**Figure 11:**
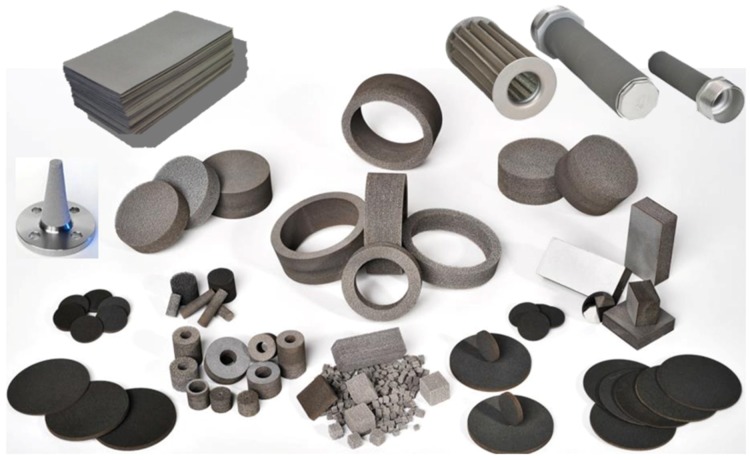
Array of products (filters) for functional applications (courtesy of Alantum).

Due to the high temperature and chemical resistance of metal foams, combined with sound absorbing properties, open cell foams from Alantum have been applied as silencers, mufflers, diesel particulate filters, selective catalysis reduction, *etc.* [28,40] (see Figure 11). Exxentis (Wettingen, Switzerland) and Mott Corporation (Farmington, CT, USA) offer filters for the filtration of gases and liquids, flow control, diffusion, sparging, fluidizing, venting and wicking [42,110]. Duocel^®^, Recemat, Inco and Alantum have open cell metal foams as a catalyst carrier, for filtration fluid control or anti-sloshing purposes in their portfolios [28,37,41,111]. Even nanoporous gold foams find application as catalysts [112].

**Figure 12:**
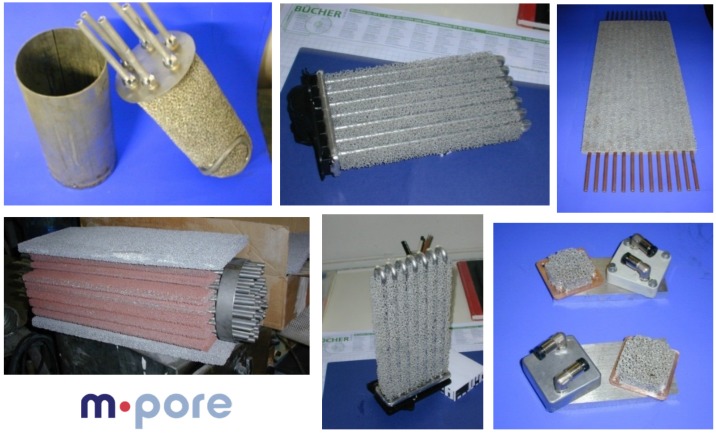
Diverse types of heat exchangers based on Al- and Cu-foams (courtesy of M-pore and Mayser GmbH).

A strong market for metallic sponges, with a large number of different products, is represented by heat exchangers. The very good thermal conductivity of metals and the large area of metal sponges provide very effective passive cooling [37,41,54,75]. As an example, Figure 11 shows a large number of filters from Alantum, Korea, and Figure 12 of different heat exchangers from M-pore, Germany [38].

ERG produces also heat exchanger from Duocel^®^ (Al- and Cu-based open cell foams) [37]. They have built different devices, e.g., a small heat exchanger to thermally stabilize the lens of electron scanning microscopes or thermal energy absorbers for medical laser applications. Furthermore, the breather plug used in the Lockheed Martin F-22 fighter aircraft for pressure releases during rapid altitude changes, electromagnetic shielding protection and moisture wicking is made of Duocel^®^ [37]. An ERG heat exchanger made of Al-foam was used as heat exchange media and support matrix of granulated chemicals consisting of multiple layers of amine-based filter beds to remove carbon dioxide and moisture in the space shuttle, and it is used now also in the International space station. After reaching its full absorption capacity, the assembly rotates to emit the CO_2_ and moisture into space, after which it can rotate back towards the interior and continue filtration, facilitating uninterrupted CO_2_ removal [113].

**Figure 13:**
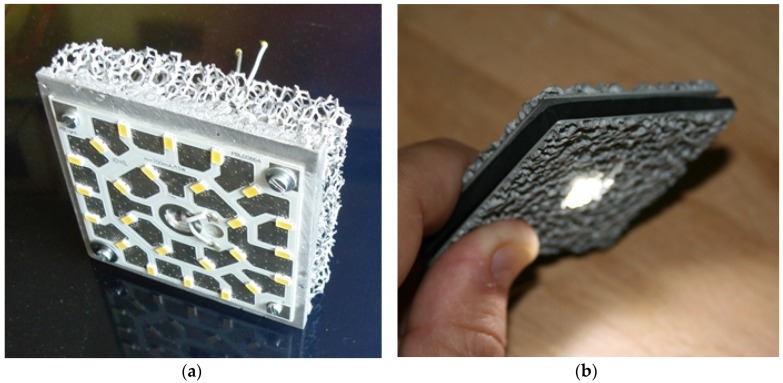
Passive thermal cooling of LED lamps: (**a**) Al open cell foam from M-pore (courtesy of M-pore); and (**b**) cut AFS from the Technical University Berlin.

A special application of heat exchangers is passive thermal cooling, a field where the demand of very effective heat sinks is increasing from day to day due to the rapidly growing performance of computers and mobile electronic devices. Nowadays, standard bulbs are being replaced by modern, powerful LED lamps (see Figure 13). This power has to be cooled down to assure LED efficiency and protect the electronics. Here, where a “noisy” fan cannot be installed, foamed passive cooling devices have an opportunity. These applications can combine their functionality with an innovative design.

Due to the high melting temperature and thermal conductivity of metals, they can be used for heat and fire resistance. Alulight provided Al-alloy foam panels for the protection of concrete against flames in fire houses, dissipating the heat over large surfaces. Similarly heat dissipation was used by Pohltec Metalfoam on AFS panels by hot forming and covered by a ceramic plasma coating as barbecue and cooking plates, providing a convenient homogeneous temperature distribution [15]. Duocel^®^ aluminum foam was used as the primary component in satellite cryogenic tanks to provide uniform heating and cooling. Space-based infrared optics using solid cryogenic coolers utilize Duocel^®^ aluminum foam also as an isothermalizer and baffle structure. Keeping a solid cryogen at a uniform temperature gives the infrared optics a longer useful lifetime [37].

Al-, Zn- and Ni-sponges are used for electro-chemical applications, where the large surface offers good advantages during operation, e.g., in water purification where ions react with the matrix material or as electrodes for Zn- and Ni-based batteries [28,39,41,111,114,115,116,117]. The application of Inco Ni-foams as the anode in NiCd and NiMH batteries comprehended a production volume of about 3,000,000 square meters of foam per year, being the most successful commercial application of metal sponges in the market.

**Figure 14:**
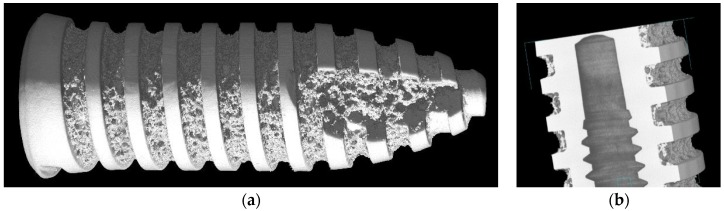
(**a**) Tomography of a Ti-based porous dental implant for osseointegration improvement; and (**b**) detail of the inner structure and porous surface (courtesy of Louis-Philippe Lefebvre).

Bio-medical applications are another field of interest. Here, high quality products based on Ti-foams provide excellent bio-compatibility properties. The trend goes into the direction of porous structures to improve osseointegration, as is shown in Figure 14, where a tomography of a Ti-based dental implant shows its porous structure. Although Ti is quite difficult to foam, foam-like structures can be created through different production methods. This field should be exploited more deeply in the future.

### 5.3. Architectural Applications

The organic cellular structure of metallic foams makes their surface unique and very attractive for design purposes. Closed cell foams with a bubbly surface, cut closed cell foams with a first open cell layer or open cell foams, which are more or less translucent depending on the pore size and thickness of the material, have become in the last decade very interesting for architectural applications. These types of applications, especially facades, are very appealing for metallic foam producers, as usually large areas are required, promising good benefits.

**Figure 15:**
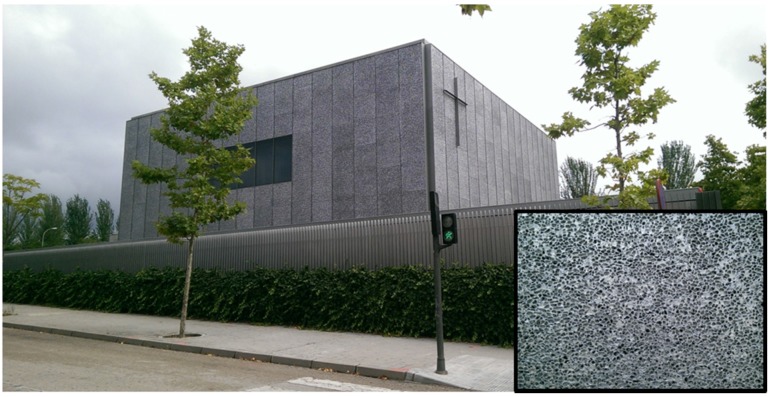
Protestant church in Terrasa, Spain, clad with Alusion foam. The inset shows a detailed view of the foam structure (courtesy of John Banhart).

In 2003, the architect Slawomir Kochanowicz covered his office building in Bochum, Germany, with closed cell Alporas Al-foam. Further facades came in the last few years, like the one of the conference center in Mallorca, Spain, made of ~20,000 m² of Cymat/Alusion Al-foam, a $2.2 million contract. Cymat also provided cladding of the exterior facade of a 12,000 m² building in downtown Tbilisi, Georgia, and recently of a Protestant church in Terrasa, Spain (see Figure 15).

**Figure 16:**
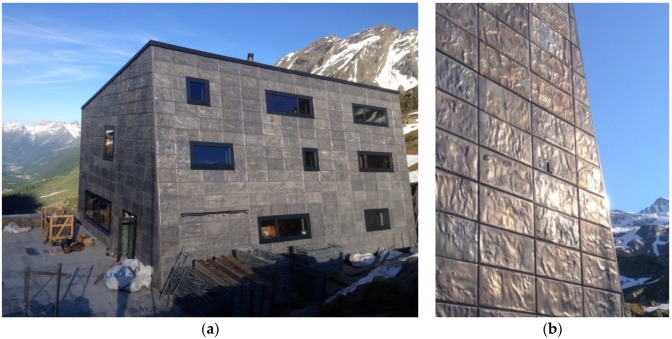
(**a**) Cottage “Anenhütte” (Lötschental, Switzerland) with AFS cladding; and (**b**) detail of the facade tiles (courtesy of Pohltec Metalfoam).

In 2015, Pohltec Metalfoam clad a cottage located in Switzerland at high altitude, with additional functionality, namely to protect the facade from deterioration due to high temperature variations and strong weather conditions (see Figure 16). The individual and organic surface of the tiles is reached by remelting the face sheet of an AFS panel.

**Figure 17:**
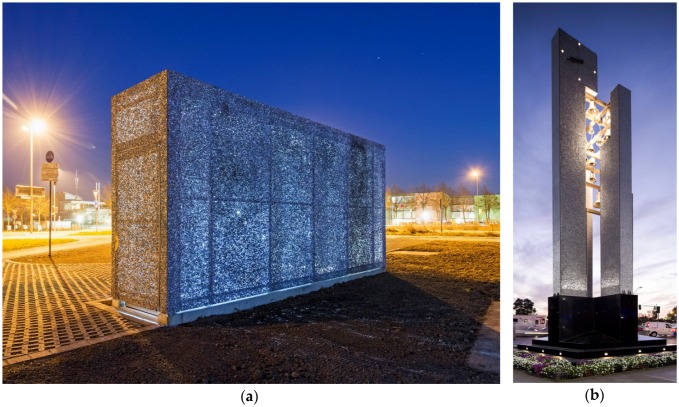
Translucent entrance of a souterrain and bell tower monument made of Alusion foam (courtesy of Alusion).

Not only facades, but other architectural applications, where realized with Alusion foam, for example the entrance of a souterrain or the support structure of a clarion as part of a bell tower monument, as shown in Figure 17a,b, respectively. Furthermore, the memorial of Service Employees International Union, dedicated to its members lost in the World Trade Centre attack of 11 September 2001, designed by the architects Furnstahl & Simon (Montclair, CA, USA) is made of Alusion foam.

**Figure 18:**
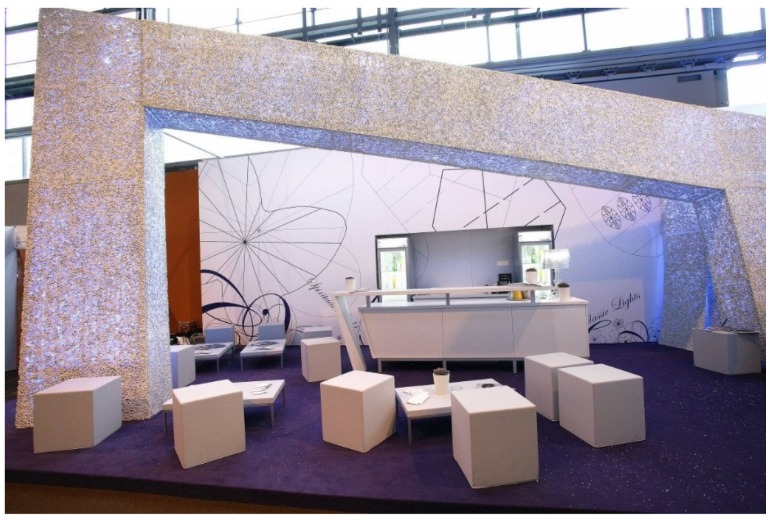
Booth for the convention center with an Alusion foam structure (courtesy of Alusion).

**Figure 19:**
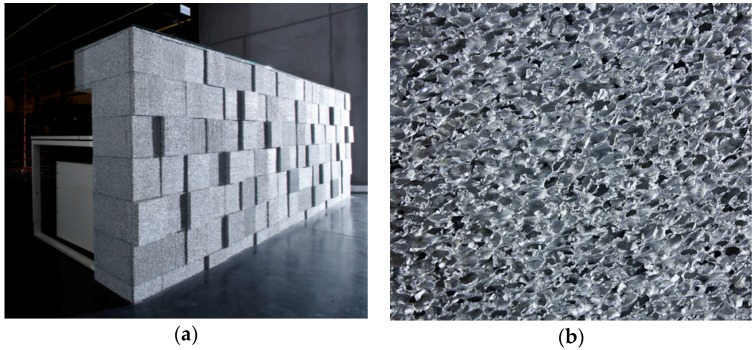
(**a**) Reception desk from Aluinvent made of Aluhab foam with a bubble size of 3 mm built for Market Zrt. Budapest, Hungary; and (**b**) detail of the foam surface structure (courtesy of Norbert Babcsán, Aluinvent).

**Figure 20:**
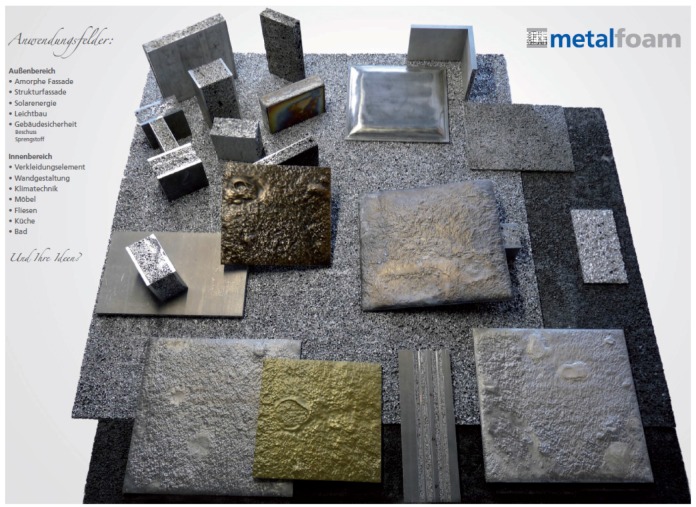
Different AFS, cut AFS and tiles made of AFS for internal architectural applications (Courtesy of Pohltec Metalfoam).

In 2003, Kauffmann, Theilig & Partner and Markgraph created for Daimler Chrysler a booth at the international auto show in Geneva, Switzerland, from Alporas foam blocks to represent their advanced technologies. Ferrari, Audi and others presented also booths with metal foam components (Figure 18). The company Aluinvent has manufactured a reception desk for Market Zrt. (Budapest, Hungary) (Figure 19). Pohltec Metalfoam has design tiles made of a molten AFS surface in their portfolio for interior architecture purposes (Figure 20).

### 5.4. Design, Art and Decoration

The classification of an application as architectural or design, art and decoration is very difficult. The transition is smooth and seamless, and it is often not possible to distinguish between them. In most cases, a combination of purposes is present; we can speak then about multi-functional applications. In this field, an immense variety of individual artworks exists, making it impossible to review all of them; therefore, only a few examples will be mentioned here. The peculiar, modern-looking and sophisticated organic surface of the cellular metal foam structure is here always in the foreground, while the other properties stay almost in the background.

**Figure 21:**
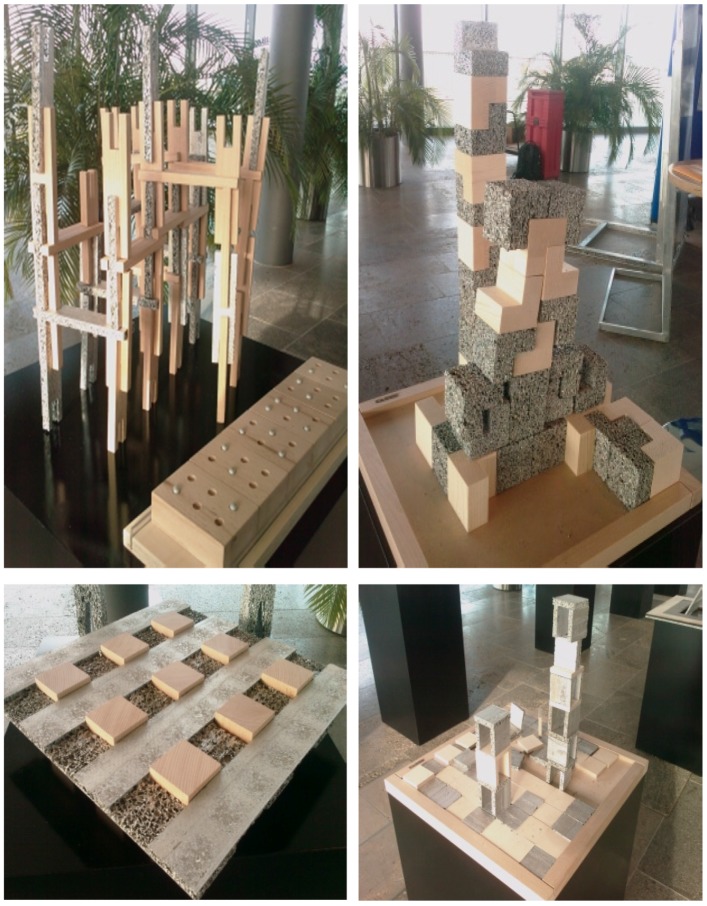
Artistic compositions of the designer Gerd Kaden created from wood and metallic foams.

Several artists and designers use metallic foams for decorations and artistic compositions. The designer Gerd Kaden produced several compositions of wood and metallic foam to show the contrast between natural and man-made cellular materials (Figure 21).

**Figure 22:**
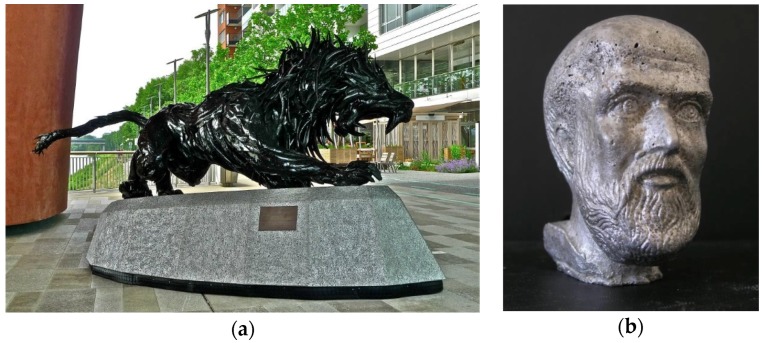
(**a**) Statue support made of Alusion foam (courtesy of Alusion); and (**b**) head of Pythagoras made of Alulight metal foam by the Slovak Academy of Sciences (courtesy of Fero Simančík, SAS).

**Figure 23:**
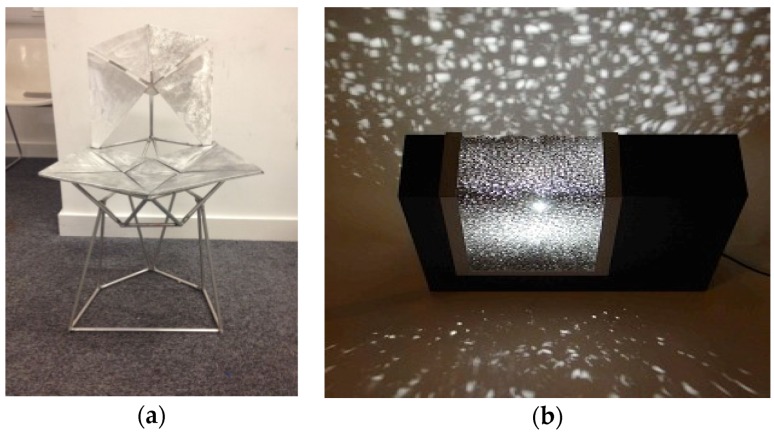
(**a**) Welded design chair made of Aluhab (courtesy of Norbert Babcsán, Aluinvent); and (**b**) art composition with metal swarm and light scattering effect from M-pore (courtesy of Mayser).

Figure 22a shows the support of a statue made of Alusion foam and Figure 22b the head of Pythagoras foamed in a mold with Alulight aluminum foam by Fero Simančík from the Slovak Academy of Science in Bratislava. A design chair from welded Aluhub foam was produced at the Imperial College London by Andor Ivan (Figure 23a). An artistic light scattering effect of a designer lamp manufactured with M-pore open cell foam can be observed in Figure 23b. Additionally, even gold and silver foams are used for jewelry, demonstrating the diversified fields of applications based on metallic foams existing on the market.

## 6. Conclusions

The technology of foamed or spongy metals is finding its place in the market; the number and volume of commercial applications is growing slowly, but step by step. Scientific research should contribute by gaining knowledge about these complex structures and supporting research and development activities, e.g., by developing new alloys, improving foam homogeneity and the production methods. Although the actual developments do not forecast a revolution in the field, almost new fields and types of applications emerge. This industrial sector, thanks to the superior and unique properties in certain applications, will continue prospering as in the past years. A combination of cost-effective production processes and quality and reproducibility improvements could accelerate the process. 
